# Early Macular Ganglion Cell Loss in Leber Hereditary Optic Neuropathy, an Optical Coherence Tomography Biomarker to Differentiate Optic Neuritis

**DOI:** 10.3390/jcm14061998

**Published:** 2025-03-15

**Authors:** Julian A. Zimmermann, Martin Dominik Leclaire, Jens Julian Storp, Tobias J. Brix, Nicole Eter, Julia Krämer, Julia Biermann

**Affiliations:** 1 Department of Ophthalmology, University Hospital Muenster, Albert-Schweitzer-Campus 1, 48149 Muenster, Germany; info@augenklinik.de (M.D.L.); jensjulian.storp@ukmuenster.de (J.J.S.); nicole.eter@ukmuenster.de (N.E.); juliabiermann2017@gmail.com (J.B.); 2 Institute of Medical Informatics, University of Muenster, 48149 Muenster, Germany; tobias.brix@ukmuenster.de; 3 Department of Neurology, University Hospital Muenster, Albert-Schweitzer-Campus 1, 48149 Muenster, Germany; julia.kraemer@ukmuenster.de; 4 Faculty of Medicine Muenster, Albert-Schweitzer-Campus 1, 48149 Muenster, Germany; 5 Department of Ophthalmology, Klinikum Bielefeld gem. GmbH, 33604 Bielefeld, Germany

**Keywords:** Leber hereditary optic neuropathy, optic neuritis, neuro-ophthalmology, optic disc, retinal nerve fibre layer, macular ganglion cells

## Abstract

**Background/Objectives**: Leber hereditary optic neuropathy (LHON) is often misdiagnosed in its early stages as idiopathic single isolated optic neuritis (SION) or multiple-sclerosis-associated optic neuritis (MS-ON) due to the young age of the patients, the subacute vision loss, and the central visual field defect. The aim of this retrospective study was to evaluate changes in the peripapillary RNFL and GCLT over time in patients with early LHON, MS-ON, and SION in order to differentiate Leber hereditary optic neuropathy (LHON) from optic neuritis (ON) in the early stages of the disease. **Methods**: Patients with LHON and ON (either idiopathic single isolated optic neuritis (SION) or ON as the first symptom of relapsing–remitting multiple sclerosis (MS-ON) were included. Optical coherence tomography (OCT) scans were reviewed. The inclusion criteria were at least one follow-up OCT examination and a definite diagnosis after examination. Changes in the peripapillary retinal nerve fibre layer (RNFL) and macular ganglion cell layer thickness (GCLT) in both groups were evaluated over time and compared with normative data. The analysis focused on the early phase (0–45 days) after symptom onset. **Results**: Nine LHON patients with early OCT scans and twenty patients with ON were included. Quantitative OCT analysis showed greater RNFL swelling in LHON compared to ON during the first 60 days after symptom onset. Between day 61 and day 120, subnormal RNFL values were observed in both groups compared to controls. Thereafter, the RNFL decreased continuously and severely in the LHON group. The RNFL of ON patients did not show a clear progression after day 120. The GCLT in five LHON eyes showed a strong and solid decrease from day 0 to day 45, which was stronger than the moderate atrophy measured in ON eyes. Continuous GCL atrophy was measured until day 121 in LHON, after which a floor effect was reached. The GCLT in the inner nasal and inner inferior sectors was significantly smaller in LHON compared to ON patients on days 0–45. **Conclusions**: Thinning of the GCLT occurs at an early stage in LHON patients. Thus, GCLT may become a diagnostic tool to differentiate LHON from ON in the early phase of disease.

## 1. Introduction

Leber hereditary optic neuropathy (LHON) is an inherited mitochondrial disease of the optic nerve, although autosomal inheritance has recently been discovered [1,2,3]. The disease follows a classic course, usually affecting the second eye within weeks to months of the onset of symptoms. It is characterised by functional visual impairment in the subacute phase mostly followed by degeneration of retinal ganglion cells and axons later on. Degeneration mainly affects the small-calibre fibres of retinal ganglion cells located at the papillofoveal bundle [4].

The m.11778G→A, m.14484T→C and m.3460G→A mutations are the most common LHON point mutations described in the literature [5]. These three hotspot mutations, which affect the nicotinamide adenine dinucleotide dehydrogenase of mitochondrial complex I of the electron transport chain, are found in approximately 90% of patients.

The estimated prevalence of LHON is approximately 1 in 45,000 people in Europe [6]. The disease affects women and men of all ages, but a significant increase in the age of onset of vision loss has been observed in males between the ages of 14 and 26 years [5].

LHON is often misdiagnosed in its early stages as idiopathic single isolated optic neuritis (SION) or multiple-sclerosis-associated optic neuritis (MS-ON) due to the young age of the patients, the subacute vision loss, and the central visual field defect [7]. While age and gender distribution do not help to differentiate between the two diseases in the early stages, both diseases often follow a stereotypical course. In contrast to LHON, SION and MS-ON are unilateral in most cases, are regularly associated with pain on eye movement, and show recovery of vision over several months with and without steroid treatment [8,9].

Funduscopic changes may be present in both conditions, most commonly mild optic disc swelling [8]. However, atypical forms of optic neuritis (e.g., as part of neuromyelitis optica spectrum disorder (NMOSD) or myelin oligodendrocyte glycoprotein antibody-associated disease (MOGAD)) are difficult to distinguish from early LHON. MOGAD and AQP4+ NMOSD should not be overlooked, as early treatment with steroids is crucial for retinal ganglion cell survival [10,11].

Clinicians are therefore faced with a major diagnostic and therapeutic challenge in early LHON and ON patients. Optical coherence tomography (OCT) of the optic nerve and macula may help to further characterise both diseases. Although current comparable data are sparse, peripapillary retinal nerve fibre layer (RNFL) thickness and macular ganglion cell layer thickness (GCLT) show different aspects in early LHON and MS-ON. While a thickened RNFL is observed in the acute stage of both diseases, there is evidence of a characteristic pattern of reduction in both RNFL thickness and GCLT as the disease progresses [12,13,14,15,16]. The aim of this study was to evaluate changes in the peripapillary RNFL and GCLT over time in patients with early LHON, MS-ON, and SION in order to differentiate Leber hereditary optic neuropathy (LHON) from optic neuritis (ON) in the early stages of the disease.

## 2. Materials and Methods

The present study was a retrospective analysis of electronic medical records (FIDUS, Arztservice Wente GmbH, Darmstadt, Germany and ORBIS, Dedalus HealthCare, Bonn, Germany) between 2016 and 2023 at the University Hospital Münster. Consecutive patients were included either with a confirmed diagnosis of LHON (n = 9) who presented to the Department of Neuroophthalmology of the Ophthalmology clinic or patients with SION (n = 8) or MS-ON (n = 12) who presented at the Department of Neurology. The inclusion criteria were at least two consecutive visits to the Department of Ophthalmology within the first year after the onset of vision loss in the affected eye. Patients with idiopathic SION and/or ON as the first symptom of relapsing–remitting MS (RRMS) diagnosed according to the revised McDonald criteria 2017 (MS-ON) were also included if follow-up OCT data were available within the first year after ON [9,17]. The diagnostic evaluation for patients with idiopathic SION and MS-ON was carried out in the Department of Neurology and included magnetic resonance imaging (MRI), lumbar puncture, visual evoked potentials, and blood tests, including antibody tests for MOG and aquaporin-4. The tests for MOG and aquaporin-4 antibodies in the serum were negative in all patients with idiopathic SION and MS-ON.

All patients were scanned with a 1.5 T Philipps MRI scanner (Eindhoven, The Netherlands). Employing the same MRI parameters and protocol for each patient, we obtained isotropic 3D T1-weighted (T1w) sequences natively and after intravenous gadolinium-diethylenetriamine penta-acetic acid injection (0.1 mmol/kg body weight), 3D fluid-attenuated inversion recovery (FLAIR) images, axial T2-weighted (T2w) images, and axial susceptibility-weighted imaging (SWI) sequences. The exclusion criteria were in general incomplete data sets, missing follow-up examinations, artefacts in OCT imaging, other ophthalmological or neurological diseases, systemic conditions that could affect the visual system, medication intake of drugs with known toxic side effects to the retina or optic nerve (e.g., chloroquine, amiodarone, or ethambutol), or unclear diagnoses. Patients with LHON were above all excluded if they indicated they were alcohol/drug-dependent or malnourished to avoid overlapping influences with toxic and nutritional optic neuropathies. Furthermore, patients with ON were excluded if their clinical and paraclinical examinations gave indications for other diseases than idiopathic SION or MS-ON [5]. The information was recorded using Microsoft Office Excel (Redmond, WA, USA; version 16.71). Data collection and data curation were performed in collaboration between two experienced neuro-ophthalmologists and one neurologist.

**Figure 1:**
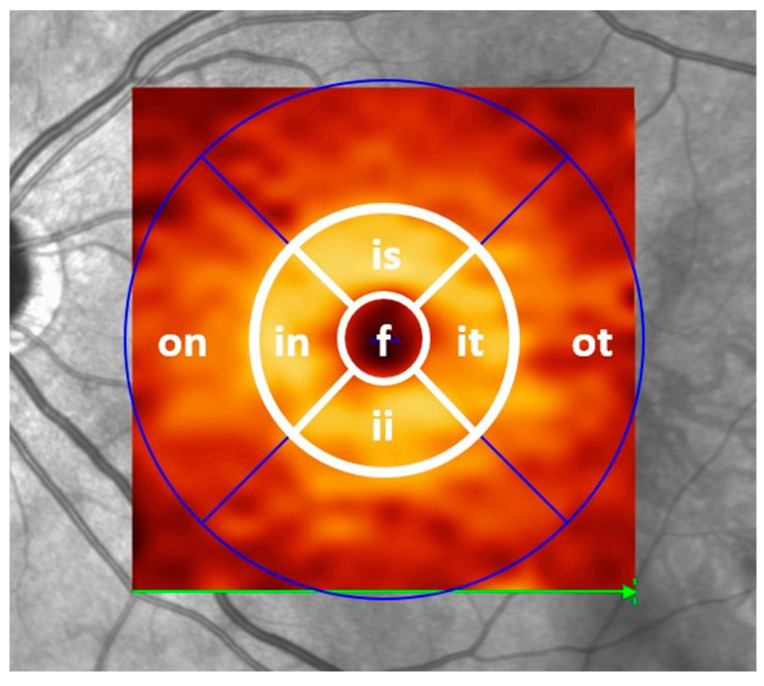
Early Treatment Diabetic Retinopathy Study (ETDRS) grid projected over the segmented macular ganglion cell layer of a healthy individual in spectral-domain optical coherence tomography (SD-OCT). Note the thick doughnut-shaped region of ganglion cells surrounding the fovea. The inner circle ganglion cell layer thickness (GCLTi) value contains the mean thickness (in µm) of the four inner sectors (in: inner nasal; is: inner superior; it: inner temporal; ii: inner inferior). The GCLT distribution per sector over the entire horizontal grid was focused on in the very early post-symptomatic phase, including the sectors on: outer nasal; f: foveal; and ot: outer temporal.

Each participant underwent a refractive eye examination, anterior segment examination, fundoscopy, and perimetry using the automated Humphrey Visual Field Analyzer II (HFA II, model 750, manufactured by Carl Zeiss Meditec AG, Jena, Germany). Specifically, the 30-2 Swedish Interactive Threshold Algorithm (SITA fast) standard programme was used for assessment. Spectral-domain OCT imaging of the macula and optic nerve head was performed using the Spectralis system (Heidelberg Engineering Ltd., Heidelberg, Germany) without pupil dilation in room light and with automatic real-time (ART) image averaging and an activated eye tracker. Analysis included automated retinal layer segmentation and thickness assessment of volumetric retinal B-scans using Heidelberg Engineering’s segmentation tool (Heidelberg Eye Explorer software version 1.9.10.0, Heyex, Heidelberg Engineering, Heidelberg, Germany). Retinal layers were automatically defined and manually adjusted if necessary. Macular data were obtained using a macular volume scan centred on the fovea. GCLT measurements were obtained from the four inner ring sectors (1–3 mm in diameter) of the Early Treatment Diabetic Retinopathy Study (ETDRS) macular thickness map (Figure 1). The measurements (in µm) of these inner sectors were summed and divided by four. This value will be referred to as the inner circle ganglion cell layer thickness (GCLTi). Peripapillary global RNFL data were obtained from circular ring scans of 12° (~3.4 mm) diameter (1536 A-scans; ART 16 to 99) placed around the optic disc. The RNFL was defined as the average of all RNFL sectors and was automatically generated by the Heidelberg system. Global RNFL and GCLTi were also calculated in 501 healthy controls who were part of a recent study at the Department of Ophthalmology [18].

Retinal layer thicknesses were reported in line with the APOSTEL 2.0 recommendations [19]. For OCT-research-specific quality assessment, we used the OSCAR-IB consensus criteria [20].

Different dates of patient visits were pooled into defined time intervals (initial findings; 0–45 days, 46–60 days; 61–90 days; 91–120 days; 121–150 days; 151–180 days; 181–270 days; 271–365 days; >366 days). OCT data from patients within these time windows were averaged.

The described research adhered to the tenets of the Declaration of Helsinki. Due to the study’s retrospective character, the University of Muenster’s Ethics Committee in North Rhine Westphalia, Germany, waived informed consent according to § 6 of the Health Data Protection Act NRW (GDSG NRW).

### Statistical Evaluation

Descriptive statistics are presented as the mean ± standard deviation (SD) or median (25th percentile; 75th percentile), depending on the distribution. GraphPad^TM^ Prism 10 software (GraphPad Software Inc., La Jolla, CA, USA) was used to perform statistical calculations and to generate graphs. Significance levels were calculated using the non-parametric Mann–Whitney U test. Significance was defined as *p* ≤ 0.05.

## 3. Results

The study included 16 eyes of nine patients diagnosed with LHON. An m.11778G→A mutation was found in eight cases and a rare m.3733G→C mutation in one case. Within seven consecutive months, subacute visual loss occurred in both eyes within a few days. Funduscopic examination revealed initial swelling of the optic disc bilaterally, which progressed to atrophy. The comprehensive neurological and ophthalmologic examination did not reveal any pathologic findings. Treatment with Idebenone resulted in an increase in visual acuity and visual field. A total of 12 eyes had a visual acuity of 1.0 LogMAR or worse at initial presentation. Eight out of the nine patients had received systemic therapy with cortisone for suspected ON at the time of initial presentation. Two patients were female, and seven were male. All but one patient developed visual loss in the other eye within one year of initial symptoms. Data on these patients are shown in Table 1.

In addition, 8 eyes of 8 patients diagnosed with unilateral SION and 12 eyes of 12 patients diagnosed with unilateral MS-ON as the first symptom of MS disease were included in the study (Table 2 and Table 3).

Patient age did not differ significantly between the three cohorts. There was a male predominance in the LHON group and a female predominance in the MS-ON group, which is consistent with real life and published demographic data. Severe visual impairment was more common in LHON patients.

**Figure 2:**
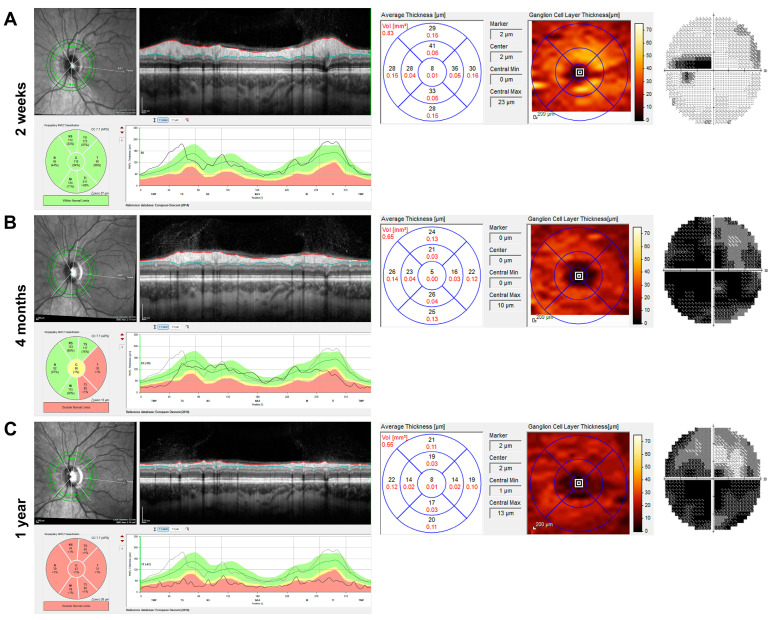
Imaging and visual field of the left eye of a 25-year-old Leber hereditary optic neuropathy (LHON) patient with m.11778G→A mutation during the first year after the onset of symptoms. Analysis of retinal nerve fibre layer (RNFL) and macular ganglion cell layer thickness (GCLT) by spectral-domain optical coherence tomography (SD-OCT, Heidelberg Engineering, Heidelberg, Germany) and Humphrey visual field (30-2, Analyser 3, Zeiss, Jena, Germany) are shown from left to right for three time points. (**A**) Two weeks after symptom onset, there was subtle swelling of the peripapillary RNFL, but already reduced GCLT in all sectors, particularly in the nasal foveal region. Centrocecal scotoma was present, and the visual acuity was 0.80 log MAR at this time. (**B**) At four months, the RNFL was partially reduced, especially in the temporal region, but there were hardly any ganglion cells visible in the colour image of the GCLT analysis. Almost complete loss of the central visual field was observed over the months. (**C**) One year after the onset of symptoms, circular atrophy of the RNFL and further reduction in the GCLT were observed with visual field depression, showing enlarged fenestration in the upper visual field.

Among the LHON patients, two patients received two OCT scans during the first year, two received three scans, two received four scans, and one patient each received five, six, and seven scans, respectively. Among the optic neuritis patients, nineteen patients received two scans, and one patient received three scans. The normally doughnut-shaped GCL showed a significant reduction in thickness soon after vision loss in LHON patients (Figure 2 shows the OCT and visual field of a representative patient two weeks, four months, and one year after vision loss), whereas the RNFL appeared preserved or supernormal in the first weeks (2A) and continuously atrophied during the first year.

Patients with LHON, SION, and MS-ON who came to our clinic between 0 and 45 days after symptom onset received their first OCT after 18 (14.5; 32) (n = 4), 10.5 (6.5; 15.5) (n = 8), and 17.5 (9.5; 30.5) (n = 4) days.

**Figure 3:**
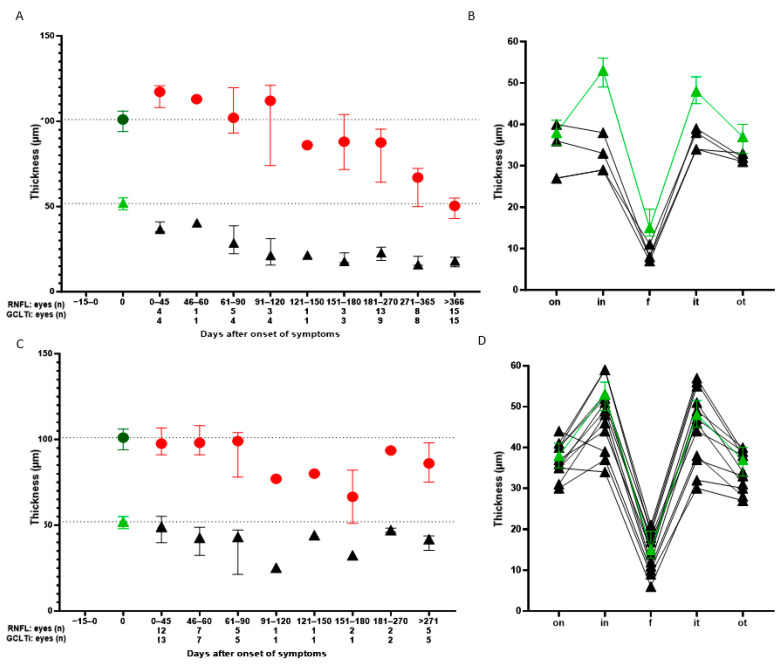
(**A**,**C**) Development of global retinal nerve fibre layer thickness (RNFL, red dots) and inner ring macular ganglion layer thickness (GCLTi, black triangles) in patients with Leber hereditary optic neuropathy (LHON, (**A**)) or optic neuritis (idiopathic single isolated ON (SION) and MS-associated ON, (**C**)). Comparison with healthy subjects (green symbols) was conducted using spectral-domain optical coherence tomography (SD-OCT, Heidelberg Engineering, Heidelberg, Germany). RNFL swelling was observed in LHON patients during the first 90 days after symptom onset. Subsequently, RNFL thickness decreased during the first year. Already in the first 45 days after symptom onset, the GCLTi is significantly reduced compared to healthy subjects (**A**). Substantial thinning of the RNFL occurs after 91–120 days in ON patients. The GCLTi is less reduced over time compared to LHON but is also noticeable in the very early phase 0–45 days (**C**). (**B**,**D**) The five horizontal GCLT sectors at 0–45 days are shown for four LHON eyes (**B**) and 13 ON eyes (**D**) (black triangles) compared to healthy subjects (green triangles). The GCLT is prematurely thinned in all LHON eyes, especially with a large delta to normative values in the inner nasal segment of the ETDRS grid (**B**). There are variable differences in ON patients compared to healthy controls (**D**). (**A**–**D**) The underlying normative values for the thickness of the global RNFL, the GCLTi, and the five horizontal sectors are taken from the study by Storp et al. based on data from 501 eyes of healthy subjects [18]. Values are presented as the median and interquartile range.

Quantitative analysis of the OCT RNFL data of all patients showed greater swelling in LHON patients compared to ON patients during the first 60 days after symptom onset (Figure 3A,C). Between days 61 and 120, subnormal RNFL values were first observed in both groups compared to controls. However, the RNFL in LHON patients was often still elevated or in the normal range. Subsequently, the RNFL in the LHON group decreased continuously and severely. The RNFL of ON patients showed no clear progression after day 120, but we have little meaningful data in the ON cohort at these later time intervals. Interestingly, GCLTi showed a large and solid decrease in five LHON patients between days 0 and 45. Continuous GCL atrophy could be measured up to day 121 in LHON, after which a floor effect of GCLTi was reached. An interim analysis of SION and MS-ON eyes showed no significant differences in median values for ON patients at days 0–45 for RNFL (97.50 vs. 96.00 µm) and GCLTi (49.00 vs. 50.75 µm) and at days 46–60 for RNFL (98 vs. 97 µm) and GCLTi (49 vs. 41.75 µm). Therefore, data from ON patients were pooled.

The detailed GCLT distribution per sector over the entire horizontal ETDRS grid in the very early post-symptomatic period (0–45 days) is shown in Figure 3B,D.

The GCLT is prematurely and significantly thinned in LHON patients, especially with a large delta in the inner nasal segment of the ETDRS grid (papillofoveal bundle area), compared to healthy subjects (Figure 3B). In ON eyes, there is considerable variation around normal values (Figure 3D).

**Figure 4:**
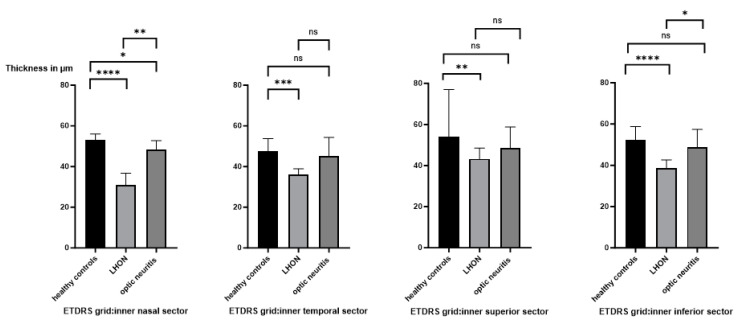
Macular ganglion layer thickness (GCLT) in the nasal, temporal, superior, and inferior inner sectors (from left to right) of the inner ring (1–3 mm diameter) of the Early Treatment Diabetic Retinopathy Study (ETDRS) macular grid in patients with Leber’s hereditary optic neuropathy (LHON), optic neuritis (ON), and healthy controls within the first 45 days after symptom onset. Compared to ON patients, there was a significant reduction in LHON eyes nasally and inferiorly. *p* value < 0.05 (*); *p* value <0.01 (**); *p* value < 0.001 (***); *p* value <0.0001 (****); not significant (ns).

Statistical evaluation of the inner nasal and inner temporal sectors is shown in Figure 4. The GCLT of the inner nasal sector of the ETDRS grid was significantly decreased in LHON compared to healthy controls and ON patients. The inner superior sector was significantly reduced in LHON patients compared to healthy controls. The inner inferior sector was significantly reduced in LHON patients compared to healthy controls and ON patients. No difference was found between healthy controls and ON patients.

## 4. Discussion

Although the courses of LHON, idiopathic SION, and MS-ON are often stereotypical, early differentiation in patients with acute monocular vision loss remains a challenge.

LHON is a rare disease that is often initially overlooked or misdiagnosed and treated as ON, especially in the early stages. This is due to several similarities between the two diseases in the majority of patients: subacute unilateral severe vision loss, young age of the patient, central scotoma in the visual field and no or only subtle fundoscopic changes. However, according to the literature, there are distinctive features. In particular, ocular pain, MRI contrast enhancement of the symptomatic optic nerve and sheath, and response to steroids are landmarks for ON [23,24]. The absence of a relative afferent pupillary defect and preserved photic blink reflex are indicative of LHON, most likely due to spared intrinsically photosensitive retinal ganglion cells [25,26,27]. However, the availability, clarity, and interpretation of the diagnostic test result, as well as the individual clinical course, often lead to an insufficient differentiation by the above criteria. A detailed medical history of relatives already affected with LHON is of course just as important as early genetic testing; however, the result of which may take weeks to months. The results of imaging and CSF studies in ON cases are crucial for early detection of optic neuritis and autoimmune optic neuropathies.

An objective imaging feature to discriminate LHON from ON as a predictive biomarker would be of great value. In this regard, early longitudinal OCT data may be helpful to visualise changes in the macula and peripapillary retina.

Several studies have shown that the inner macular thickness thins before the RNFL in patients with LHON, although the studies used different OCT devices and calculated different segmentation layers (thickness of the GCL with or without the inner plexiform layer (IPL) or total macular thickness) [14,15,28]. Since LHON is a mitochondrial disease, we believe it is more accurate to look at the ganglion cell body layer separately. The time points of analysis during the course of the disease are also variable, and data from the very early phase seemed particularly appropriate. Balducci and colleagues found thinning of the macular ganglion cell inner plexiform layer (GCIPL) in the nasal inner ring of two patients in the presymptomatic phase (6 weeks before onset of visual loss) [28]. Our analysis of LHON patients who underwent GCLT measurements within the first 45 days of symptom onset also showed early and significant thinning compared to controls. The most significant decrease in GCLT compared to healthy subjects occurred in the inner nasal and inferior sector of the ETDRS grid (Figure 3 and Figure 4). This is not surprising given the anatomical and physiological characteristics of this region: small-calibre, unmyelinated optic nerve fibres of the papillofoveal bundle, high energy demand, and high mitochondrial deoxyribonucleic acid content. Carbonelli and colleagues observed OCT structural events that marked the transition from presymptomatic to affected status in four LHON patients. In their cases, the GCL did not undergo significant changes until the onset of visual loss. After the onset of symptoms, a significant reduction in thickness was observed. Interestingly, they reported the greatest and most rapid atrophy in the nasal sectors during the first month after onset. This is consistent with our findings [29].

Thus, especially the inner nasal and inner inferior sector of the GCLT may become a diagnostic tool to differentiate LHON from ON in the early stages of the disease. Balducci and colleagues also described the natural history of GCIPL thinning in four patients over 12 months (Cirrus HD-OCT). Month 6 represented the end of GCIPL loss in their study and in a ganglion cell complex analysis performed by others [28,30]. In our cohort, GCLT decreased until approximately four months after symptom onset and then reached a relatively stable plateau (Figure 3).

In the acute stage of LHON, there is peripapillary swelling of the RNFL, which in our patients progresses to the subsequent atrophic stage within three to nine months (Figure 3). Pathological axonal swelling is considered a subclinical sign of the disease in asymptomatic LHON carriers, which may progress to pseudo-oedema and visual loss in the acute phase of the disease. In 2022, Carbonelli et al. described a consistent structural event that marked the transition from presymptomatic to affected status in four LHON patients. Despite using different OCTs, they found peripapillary RNFL thinning of the temporal sectors, which always occurs when the adjacent temporal-superior and temporal-inferior sectors increase in thickness [29].

The swelling most likely represents a compensatory increase in mitochondrial biogenesis and/or axonal stasis and reflects the above-normal RNFL thickening on OCT in the early symptomatic period [31]. As ganglion cell apoptosis progresses, atrophy and thinning of the RNFL becomes measurable. This is consistent with other studies describing RNFL reduction in LHON patients from 3 to 6 months [15,32].

What do we know about longitudinal OCT data in SION and MS-ON? ON as an inflammatory attack of the retrobulbar optic nerve sheath is a common early manifestation of MS. OCT quantification of neuroaxonal integrity by RNFL and GCIPL thickness has been extensively studied in MS patients with and without ON and in SION [13,33,34,35,36].

Increased peripapillary RNFL thickness is detectable within the first few weeks after the onset of acute ON, which may reflect inflammatory cell infiltration, interstitial oedema, blood–retinal barrier disruption, and glial cell proliferation. As the acute inflammatory process subsides, retrograde degeneration results in loss of axons in the peripapillary RNFL and loss of retinal ganglion cell bodies in the GCIPL. Dynamic changes in retinal layer thickness after acute ON appear to be limited to the first 4–6 months, after which further longitudinal changes in thickness between the ON eye and the other or non-ON eye in MS are similar. Faster thinning of the inner retinal layers has been demonstrated in the early stages of disease, along with a plateau effect with longer disease duration [36,37].

A recent review and meta-analysis of data from cross-sectional cohorts showed peripapillary RNFL thinning in MS-ON eyes (mean difference −20.10 μm) and in MS non-ON eyes (−7.41 μm) compared with control eyes, and macular GCIPL atrophy of −16.42 μm in MS-ON eyes and −6.31 μm in MS non-ON eyes [35].

This differs from LHON, as mentioned above, where the degeneration can largely be seen as a continuum. In contrast, the RNFL thickness was significantly higher in the acute phase of ON (and LHON, reflecting RNFL swelling), and the time to first detectable atrophy compared to baseline in the other eye was reported to be 1.64 months [13]. Although we analysed a similarly small number of ON patients, no continuous RNFL reduction was detectable in our patients after approximately three months of vision loss. This result is in line with what would be expected after an episode of ON has been overcome. The confounding effect of inflammation and swelling of the RNFL in early ON is avoided by using the GCLT. No swelling of the GCIPL was found in the acute phase of the disease, and it appeared unchanged compared to the unaffected contralateral eye using the Cirrus HD-OCT [16]. In their study, the GCIPL showed significant thinning 3 and 6 months after symptom onset, representing permanent neuronal loss following RGC apoptosis [16]. As seen in our data, during the early phase of ON (0–45 days), there were no significant differences in the GCLT compared to controls (Figure 3 and Figure 4), but differences were already present in LHON patients. Thus, early macular ganglion cell loss in LHON may be a diagnostic tool to differentiate LHON from MS-ON/SION.

Looking at our demographic data, it is clear that our sample reflects the typical characteristics of the three diseases in terms of gender and age. Patient age did not differ significantly between the three cohorts, as both LHON and ON manifest predominantly between the second and fourth decade of life. There was a predictable male predominance in the LHON group (70% of our patients) and a female predominance in the MS-ON group (67% of our patients), while no gender predisposition was seen in SION patients, which is consistent with real life and published demographic data. Severe visual impairment was more common in LHON patients. However, visual acuity does not appear to be a suitable distinguishing criterion, as it was heterogeneous in all three groups and depends on the stage of disease.

The results presented in our study must be interpreted with caution. First, it is a retrospective cohort study, which in itself is a source of bias and confounding. As a result of the retrospective design, there were no fixed appointments with patients for follow-up, and therefore, we had to pool the data into time intervals. Since LHON is a rare disease, a larger number of patients would be desirable. A history of heavy drinking and smoking or concomitant medication was not known in our patients. However, we could not exclude the possibility that any of these aspects could influence and modify the pattern of RGC loss seen in our patients. Future prospective studies with standardised follow-up intervals are needed to analyse retinal degeneration in LHON and patients with different types of autoimmune ON as soon as possible to confirm our findings.

Finally, further evaluation is needed to differentiate early GCLT in LHON patients from other optic nerve and neurological diseases to address the specificity and sensitivity of this potential diagnostic marker. LHON may be associated with multiple comorbidities, in particular several demyelinating diseases of the central nervous system (LHON-plus) [38,39]. Among the LHON-plus disorders, LHON–multiple sclerosis (MS) has been reported in the literature [38]. Furthermore, ON in NMOSD or MOGAD may have different characteristics in macular segmentation analysis. Syc et al. reported that GCL-IPL, RNFL, and mean macular thickness were significantly reduced in eyes with neuromyelitis optica compared to MS-ON eyes and non-ON MS eyes [16]. This is an interesting topic for future research.

## 5. Conclusions

In summary, a common way to differentiate LHON from MS-ON/idiopathic SION is still the response of MS-ON/idiopathic SION patients to high-dose cortisone treatment (“ex juvantibus”) and the unalterable progressive vision loss in LHON patients. While ON patients usually show a rapid improvement in symptoms, LHON patients do not. Unfortunately, this leads to delayed diagnosis and mismanagement of LHON patients. Early OCT GCLT reduction in LHON may provide a diagnostic biomarker after the onset of vision loss in young patients to differentiate LHON from MS-ON/idiopathic SION. Future studies in additional cases are needed to further establish the importance of GCLT assessment in the management of LHON.

## Figures and Tables

**Table 1:** Summary of LHON patient demographics (data for continuous variables are presented as mean (±standard deviation) or median (25th percentile; 75th percentile), depending on the data distribution). * m3733G→C: rare mitochondrial DNA mutation in the ND1 gene, previously described in patients with LHON [21]. Visual acuity and severity grading were based on conversion tables by Michael Bach [22].

LHON Patients	Females	Males
Number of patients	2	7
Number of eyes	4	12
Age at onset of symptoms (years)	38.92 ± 13.19	38.75 ± 15.16
Mutations (number of patients)		
m.11778G→A	2	6
m.3733G→C *		1
Bilaterality within the first year after initial symptoms (number of patients)	2/2	6/7
Visual acuity at first presentation in the affected eye (LogMAR) (n)		
0.00–0.40 (no–mild impairment)	1	2
0.50–1.30 (moderate–severe impairment)	3	9
1.40–1.70 (blindness)		1
1.80–2.40 (off-chart visual acuity)		

**Table 2:** Summary of patient demographics in idiopathic single isolated optic neuritis (SION): Data for continuous variables are presented as mean (±standard deviation) or median (25th percentile; 75th percentile), depending on the data distribution.

SION Patients	Females	Males
Number of patients	4	4
Number of eyes	4	4
Age at onset of symptoms (years)	37.25 ± 11.45	31.50 ± 5.50
Visual acuity at first presentation in the affected eye (LogMAR) (n)		
0.00–0.40 (no–mild impairment)	2	1
0.50–1.30 (moderate–severe impairment)	2	1
1.40–1.70 (blindness)		1
1.80–2.40 (off-chart visual acuity)		1

**Table 3:** Summary of multiple-sclerosis-associated optic neuritis (MS-ON) patient demographics: Data for continuous variables are presented as mean (± standard deviation) or median (25th percentile; 75th percentile), depending on the data distribution.

MS-ON Patients	Females	Males
Number of patients	8	4
Number of eyes	8	4
Age at onset of symptoms (years)	30.50 ± 8.88	33.50 ± 11.08
Visual acuity at first presentation in the affected eye (LogMAR) (n)		
0.00–0.40 (no–mild impairment)	3	1
0.50–1.30 (moderate–severe impairment)	4	1
1.40–1.70 (blindness)		
1.80–2.40 (off-chart visual acuity)	1	2

## Data Availability

J.A.Z., J.K., and J.B. had full access to the data used in the analyses in the manuscript. The authors take full responsibility for the data, the analyses and interpretation, and the conduct of the research; they had the right to publish any and all data. The data sets generated and/or analysed are available from the authors (J.A.Z., J.K., or J.B.) upon reasonable request.
